# Retraction: ω3-PUFAs Exert Anti-Inflammatory Activity in Visceral Adipocytes from Colorectal Cancer Patients

**DOI:** 10.1371/journal.pone.0343572

**Published:** 2026-02-25

**Authors:** The PLOS One Editors

After this article [1] was published, concerns were raised regarding results presented in Figs 1 and 3. Specifically,

- The Fig 1A STAT3 NW band appears similar to the Fig 1B Lamin B ObCC band when flipped horizontally and the Fig 1C GAPDH ObCC band when flipped horizontally.
- The Fig 1A STAT3 Ob band appears similar to the Fig 1B Lamin B NWCC band when flipped horizontally and the Fig 1C GAPDH NWCC band when flipped horizontally.
- The Fig 1A STAT3 NWCC band appears similar to the Fig 1A STAT3 ObCC band, the Fig 1B Lamin B Ob band when flipped horizontally, and the Fig 1C GAPDH Ob band when flipped horizontally.
- The Fig 3A STAT3 NW lanes 1–2 bands appear similar to the Fig 3A STAT3 NWCC lanes 5–6 bands.
- There appear to be vertical discontinuities in the following panels:
  - Fig 3A Pstat3
  - Fig 3A STAT3

The corresponding author stated that the data underlying [1] are no longer available and that some of the above listed blots were selected as representative of the entire experiment.

In light of the above unresolved concerns which call into question the integrity and reliability of [1], the *PLOS One* Editors retract this article.

MDA, BS, RV, CS, and RM did not agree with the retraction. SG, MLF, AV, AI, CG, and SG either did not respond directly or could not be reached.

MDA, BS, RV, CS, and RM stand by the article’s findings.

## References

[1] D’Archivio M, Scazzocchio B, Giammarioli S, Fiani ML, Varì R, Santangelo C, et al. RETRACTED: ω3-PUFAs exert anti-inflammatory activity in visceral adipocytes from colorectal cancer patients. PLoS One. 2013;8(10):e77432. doi: 10.1371/journal.pone.0077432 24116229 PMC3792028

